# Gut Microbiota and Metabolic Dysfunction-Associated Steatotic Liver Disease: From Dysbiosis to Metagenomic Insights and Therapeutic Perspectives

**DOI:** 10.3390/ph19071113

**Published:** 2026-07-19

**Authors:** Otilia Elena Frăsinariu, Violeta Ștreangă, Aniela Luminița Rugină, Dana Elena Mîndru, Teodora Cristina Vintilă, Oana Viola Bădulescu, Iris Bararu-Bojan, Vasile Valeriu Lupu, Ancuța Lupu, Adriana Mihai, Isabela Ioana Loghin, Daniela Eugenia Popescu, Dragoș Florin Teșoi

**Affiliations:** 1 Grigore T. Popa University of Medicine and Pharmacy, 700115 Iași, Romania; frasinariu.otilia@umfiasi.ro (O.E.F.);; 2 Endocrinology Department, Saint Spiridon County Hospital, 700111 Iași, Romania; 3 Department of Obstetrics-Gynecology and Neonatology, “Victor Babeș” University of Medicine and Pharmacy, 300041 Timișoara, Romania

**Keywords:** dysbiosis, gut–liver axis, gut microbiota, pediatric MASLD, metabolic dysfunction

## Abstract

Metabolic dysfunction-associated steatotic liver disease (MASLD) has emerged as the most common chronic liver disorder in the pediatric population, closely paralleling the global rise in childhood obesity. Increasing evidence highlights the gut–liver axis as a key contributor to MASLD pathogenesis, with gut microbiota dysbiosis influencing hepatic steatosis through multiple interconnected mechanisms, including increased intestinal permeability, endotoxemia, altered bile acid metabolism, and modulation of host energy homeostasis. In children, the characterization of microbiota signatures associated with MASLD remains challenging due to heterogeneity across studies, age-related microbial dynamics, and methodological variability. This review synthesizes current evidence regarding the role of the gut microbiota in pediatric MASLD, focusing on pathogenetic pathways, reported microbial patterns, and microbiota-targeted therapeutic strategies, while incorporating relevant mechanistic evidence from adult studies where pediatric data remain limited. Although several taxa have been repeatedly associated with pediatric MASLD, findings are not yet sufficiently consistent for clinical application. Interventions such as probiotics, prebiotics, and dietary modulation show promising but still preliminary results, with limited high-quality pediatric trials available. A deeper mechanistic understanding and standardized study designs are needed to clarify causality and to support microbiota-based precision approaches in pediatric MASLD management.

## 1. Introduction

Metabolic dysfunction-associated steatotic liver disease (MASLD) is the most common chronic liver disease, affecting 10–20% of the pediatric population [1]. The worldwide prevalence of MASLD among patients over 15 years old is estimated to reach 33.5% by 2030 [2]. More recent meta-analyses confirm the continuous rise in prevalence, reporting global MASLD rates of approximately 32–38% in the general adult population after 2021, with even higher figures among individuals with obesity or type 2 diabetes [3,4]. In the pediatric populations, MASLD prevalence continues to rise in parallel with childhood obesity, and recent meta-analyses indicate that the disease affects a substantial proportion of children and adolescents, particularly those with overweight or obesity, in whom prevalence may exceed 40% [5,6,7]. Consequently, MASLD has become one of the most frequent liver disorders in children with metabolic risk factors, contributing to the early development of cardiometabolic complications and advanced liver disease later in life [8].

An increased number of microorganisms that reside in the gut have a close relationship with the homeostasis and immunity of the human organism. The development of the intestinal microbiome is influenced by gestational age, type of delivery, feeding modality and administration of antibiotics during the newborn period. Dysbiosis is correlated with multiple pediatric pathologies, including obesity, allergies, inflammatory bowel diseases, and MASLD [9]. Since the initial studies establishing a connection between dysbiosis and MASLD were published, there has been ongoing scientific inquiry into whether dysbiosis is a consequence of the metabolic alterations associated with MASLD or if it is a contributing factor in its development. Recent research published after 2021 increasingly supports the notion that gut dysbiosis plays a role in metabolic disorders, such as obesity and MASLD, through mechanisms that include heightened intestinal permeability, translocation of endotoxins, modifications in bile acid metabolism, and variations in microbial metabolites that influence hepatic lipid accumulation and inflammation [4,7]. However, despite growing evidence linking dysbiosis and MASLD, it remains unclear whether microbiota alterations are a primary driver of disease development or a consequence of the metabolic and inflammatory changes associated with hepatic steatosis.

This review aims to summarize the evolving understanding of the gut microbiota in the pathogenesis of hepatic steatosis, tracing the shift from early associative studies to current insights derived from metagenomic analyses and the development of targeted microbiota-modulating therapies.

Although significant advances have been made in elucidating the role of the gut microbiota in metabolic liver disease, much of the current mechanistic understanding derives from adult cohorts and experimental animal models. Pediatric-specific studies remain comparatively limited because of the lower disease prevalence, ethical constraints related to invasive sampling, and the dynamic developmental changes in the intestinal microbiome during childhood. Consequently, several pathogenic mechanisms discussed in this review are extrapolated from adult evidence and should be interpreted with caution until confirmed in pediatric populations.

## 2. Literature Search and Evidence Synthesis

This narrative review was developed through a structured literature search designed to identify the most relevant evidence regarding the role of the gut microbiota in pediatric MASLD. Electronic searches were conducted using PubMed/MEDLINE, Scopus, and Web of Science for studies published up to March 2026. To ensure comprehensive coverage of the rapidly evolving literature, additional relevant publications were identified through manual screening of the reference lists of eligible articles and recent review papers. The search strategy combined Medical Subject Headings (MeSH) and free-text terms related to gut microbiota, dysbiosis, microbiome, pediatric metabolic dysfunction-associated fatty liver disease (MAFLD), MASLD, nonalcoholic fatty liver disease (NAFLD), non-alcoholic steatohepatitis (NASH), metabolic dysfunction-associated steatohepatitis (MASH), metagenomics, metabolomics, gut–liver axis, intestinal permeability, bile acids, short-chain fatty acids, probiotics, prebiotics, synbiotics, postbiotics, fecal microbiota transplantation, and microbiota-targeted therapies. Various combinations of these terms were adapted according to the indexing system of each database. Preference was given to recent systematic reviews, meta-analyses, randomized controlled trials, prospective cohort studies, clinical practice guidelines, and high-quality mechanistic investigations published in peer-reviewed journals. Because pediatric-specific evidence remains relatively limited, landmark adult clinical studies and well-established experimental animal models were also included whenever they provided mechanistic insights relevant to pediatric disease. Throughout the manuscript, findings originating from adult populations or animal studies are interpreted with appropriate caution. Owing to the narrative nature of this review, a formal systematic review methodology and quantitative risk-of-bias assessment were not performed. Nevertheless, the evidence synthesis prioritized studies according to their methodological rigor, scientific impact, clinical relevance, and applicability to pediatric MASLD. Particular emphasis was placed on publications from the last five years to reflect the rapidly evolving understanding of gut microbiota, metagenomics, and microbiota-targeted therapeutic strategies.

## 3. Pathophysiological Mechanisms of MASLD

### 3.1. Multifactorial Theory

The pathogenesis of MASLD has been explained by multiple hypotheses over time. Initially, it was described by the “two-hit hypothesis” [10,11], in which the first hit results from insulin resistance, causing hepatic steatosis [12]. The accumulation of hepatic fat sensitizes the liver, inducing inflammation and cell apoptosis. Fat accumulation in the liver is associated with a high-calorie diet, sedentary lifestyle, and genetic predisposition [13].

Subsequently, the second hit involves the effects of inflammatory cytokines, adipokines, and mitochondrial destruction, and it promotes oxidative stress. These processes are also influenced by changes in the intestinal microbiota. These aspects lead to fibrosis and steatohepatitis, and, over time, can induce liver cirrhosis [14].

Currently, the widely accepted explanation is the more complex ‘multiple parallel hits hypothesis’, which replaces the earlier ‘two-hit’ model.” [15]. Among the multiple parallel hits, oxidative stress is regarded as the main factor contributing to the development of liver damage and disease progression. Oxidative stress refers to conditions produced by reactive oxygen species. It is defined as an imbalance between oxidants and antioxidants, in favor of oxidants, which have pathogenic and potentially destructive effects. At high concentrations, specific reactive oxygen species cause oxidative changes in cellular macromolecules (proteins, lipids, DNA), thus causing the accumulation of these damaged macromolecules and subsequently leading to liver damage [16]. The contributors to oxidative stress in liver disorders are categorized as exogenous factors—such as viruses, alcohol, and drugs—and endogenous factors, including insulin resistance, obesity, and diabetes [17].

Another important factor involves the intestinal microbiome. An unbalanced gut microbiota can lead to the production of fatty acids in the intestine, which increases intestinal permeability and, consequently, the absorption of these fatty acids. Their absorption leads to high circulating levels of molecules that contribute to the activation of inflammatory pathways and the release of pro-inflammatory cytokines such as interleukin 6 (IL-6) and tumor necrosis factor alpha (TNF-alpha) [18].

In genetically or epigenetically predisposed individuals, metabolic and environmental factors promote hepatic fat accumulation and inflammatory responses, leading to hepatocyte apoptosis, activation of hepatic stellate cells and fibrogenesis. MASLD is presently recognized as a multifactorial condition instead of a straightforward sequential progression from steatosis to inflammation [15,16].

The development of MASLD, particularly in children, is characterized by complex interactions between resident liver cells and recruited cells such as T cells and hepatic stellate cells. These interactions promote disease progression, along with other inflammatory cell-derived factors that are released either as a direct result of hepatic steatosis, hepatocyte injury and apoptosis, or as an indirect response to liver injury and/or gut-derived bacterial products acting on Toll-like receptors (TLR) [19]. Dysregulation of pro-inflammatory cytokines and adipokines is almost universally detected in pediatric patients with MASLD. Furthermore, mitochondrial oxidative stress and hepatocyte apoptosis also contribute to the development of these pathologies [20]. Hepatic stellate cells are considered the main extracellular matrix-producing cells during the development of steatohepatitis and are activated following hepatocyte injury and apoptosis [21]. Hepatic progenitor cells (the population of stem cells resident in the liver) have been shown to be involved in pediatric non-alcoholic steatohepatitis. They appear to play a role in the liver’s response to oxidative stress, and their levels may be correlated with fibrosis and disease progression. Moreover, they can undergo an epithelial–mesenchymal transition, resulting in a profibrogenic myofibroblast-like cell population [20,22].

The Kupffer cells are important regulators of biological exchanges between hepatocytes and other liver cells. They support the action of neutrophils, killer T lymphocytes and macrophages, as well as the phagocytic and removal of microorganisms. Evidence suggests that they play various roles in the pathogenesis and progression of non-alcoholic steatohepatitis [19].

There are numerous changes in the functionality of peripheral T cell subpopulations. Studies have reported a predominance of CD8+ T cells over CD4+ and CD20+ subpopulations, in association with increased levels of neutrophils [19,23].

### 3.2. The Gut–Liver Axis

The gut–liver axis represents a bidirectional relationship between the gastrointestinal tract, its microbiota, and the liver. This results from the transfer of microbiota-associated molecules and the integration of signals generated by dietary, genetic, and environmental factors [24,25,26]. Components of this axis include the intestinal barrier, the microbiota, and the liver. Reciprocal interaction occurs via the portal vein, which allows direct transport of intestinal-derived products to the liver, as well as a feedback route from the liver to the intestine. Gut–liver axis disturbances can occur due to the alteration of intestinal permeability and the composition of the intestinal microbiota [27]. The intestinal barrier is a complex functional unit, composed of luminal and mucosal elements, such as the epithelial cell layer, mucosal barrier, and components of innate immunity. It also includes the neuroenteric, vascular, and endocrine systems, digestive enzymes, and the gut microbiota. This barrier plays a key role in protecting against enteric microorganisms, potentially harmful toxins and bacterial products closely associated with susceptibility to certain pathologies. The intestinal epithelium consists of a single layer of columnar cells, which are mainly absorptive cells (80%), with the remaining 20% being Paneth, goblet, and enteroendocrine cells. The transport of molecules between intestinal cells is achieved through junctional complexes. The most important are tight junctions (TJ), adherens junctions and desmosomes. In addition to providing a barrier against harmful molecules, TJ can also function as pores which allow the passage of ions, substances, and water. Homeostasis of the intestinal epithelium requires coordination between TJ proteins, the actin cytoskeleton, endocytosis, and intracellular signaling pathways [28]. The TJ structure is constantly remodeled under the influence of external stimuli, such as food residues, pathogenic bacteria, or commensal intestinal bacteria [29,30].

In addition to regulating paracellular permeability, the intestinal barrier can activate innate immune cells (e.g., dendritic cells), thereby preventing systemic infections triggered by intestinal microorganisms [31,32]. Homeostasis of the intestinal tract is also maintained by Paneth cells, a type of specialized secretory epithelial cell found on the mucosal surface of the small intestine that produces large amounts of defensins and antimicrobial peptides [33].

Another important element of maintaining intestinal barrier homeostasis is the mucosal barrier, which is made up of a set of elements involved in achieving the physical and immunological barrier function. The intestinal mucus produced by the goblet cells consists of two layers: an outer layer that contains the commensal intestinal microbiota, antimicrobial proteins and immunoglobulin A (IgA) secretory molecules, and an inner layer with a protective role [34]. The mucus layer is the first line of defense against external molecules and prevents bacteria from attaching to epithelial cells. Intestinal microorganisms have developed several strategies over time to circumvent these mechanisms, more specifically the release of mucolytic enzymes, inhibition of mucin synthesis, and damage to tight junctions [34].

Disruption of the intestinal barrier allows the translocation of potentially pathogenic intestinal bacteria and their products, such as endotoxins, into the portal bloodstream. Impairment of the intestinal barrier, increased intestinal permeability, and bacterial overgrowth in the small intestine appear to play an essential role in the pathogenesis of MASLD [35].

### 3.3. Dysbiosis

The intestinal microbiota consists of billions of organisms with numerous species. Gut microbiota dysbiosis refers to an imbalance in the microbial community, causing qualitative and quantitative changes [36]. The human intestinal microbiota mainly consists of Bacteroidetes and Firmicutes. Proteobacteria, Verrucomicrobia, Actinobacteria, Fusobacteria, and Cyanobacteria are present in much smaller proportions. Physiologically, they contribute 5–15% to providing energy from food by fermenting undigested food residues [37]. Alterations in the gut microbiota of obese individuals are associated with enhanced metabolic activity, increased energy extraction and activation of lipogenic and inflammatory pathways that contribute to hepatic fat accumulation [38]. The intestinal microbiota has been implicated in both gastrointestinal disorders and a wide range of extraintestinal conditions, including obesity, type 2 diabetes, cardiovascular diseases, and other metabolic disorders [39].

Hepatotoxic bacterial products, including pathogen-associated molecular patterns (PAMPs) and damage-associated molecular patterns (DAMPs), can reach the liver through the portal circulation. These molecules activate pattern recognition receptors, particularly TLRs, expressed on Kupffer cells, hepatic stellate cells, and hepatocytes. Lipopolysaccharide, a component of the cell wall of Gram-negative bacteria, is one of the best-characterized PAMPs and a key ligand for TLR4, initiating pro-inflammatory signaling cascades and promoting the production of cytokines such as TNF-α. Activation of TLR-mediated pathways contributes to hepatic inflammation, stellate cell activation, and fibrogenesis, thereby playing a significant role in the progression of MASLD. In addition, other TLRs, including TLR2, can recognize bacterial components and further modulate intestinal permeability and immune responses, linking gut dysbiosis to liver injury [40,41].

Inflammasomes (intracellular, multiprotein complexes with a central role in innate immunity) detect and respond to a wide range of PAMPs. This process leads to their activation and subsequently to the maturation of pro-inflammatory cytokines such as IL-1 and IL-18. This activation, which is crucial in host defense against pathogens, also appears to play an important role in the inflammatory component of obesity and MASLD [42].

Numerous factors, including diet, lifestyle, medications, host genetics, and environmental exposures, can influence the composition and function of the intestinal microbiota. These factors contribute to the interindividual variability of the gut microbiome and its metabolic and immunological effects on the host [43]. Conversely, the gut microbiota influences the metabolic phenotype of the host, participates in food and drug metabolism, and can enhance the immune system [44].

Host genetic factors have been shown to influence the composition and function of the gut microbiota. Genome-wide association studies have identified multiple human genetic loci associated with specific bacterial taxa and microbial metabolic pathways, highlighting the role of host genetics in shaping microbial communities and host–microbe interactions [45]. Variants in host immune-related genes, particularly those involved in innate immune sensing such as nucleotide-binding oligomerization domain-containing protein 2 (*NOD2*), have been associated with alterations in gut microbiota composition. These genetic differences can influence microbial diversity and the abundance of specific bacterial taxa, highlighting the role of host immune genetics in shaping intestinal microbial communities [46]. Under conditions of homeostasis, the host and the microbiome benefit from each other through a state called eubiosis. Conversely, a disturbance in the structure or function of the microbiome, known as dysbiosis, results from an abnormal ratio of commensal and pathogenic bacterial species.

Germ-free animal models have long been used to define the consequences of the absence of gut microbiota and, therefore, to establish which physiological functions of the host are influenced by these bacteria. Among these functions, the gut microbiota has been shown to play a role in obesity and the development of metabolic diseases [47]. Germ-free animals were found to be resistant to obesity induced by various diets, including high-fat, Western-style, or high-sugar diets. This resistance was associated with increased angiopoietin-like 4 (*ANGPTL4*) gene expression, higher AMP-activated protein kinase (AMPK) activity, and changes in downstream targets such as acetyl-CoA carboxylase (ACC) [48]. However, the obesity-resistance phenotype in these animal models strongly depends on the sugar composition of the diet [48].

Fleissner et al. studied the effects of three different diets (low fat, high fat, and Western diet) on conventional and germ-free animal models. The study did not reach a consensus on the role of *ANGPTL4* in protecting germ-free rodents against obesity. Notably, both groups showed no difference in body weight gain on the low-fat diet, but germ-free rodents gained more body weight and total fat than conventional rodents on the high-fat diet. Conversely, germ-free rodents fed a Western diet had significantly less body fat, suggesting that the absence of gut microbiota does not generally protect against diet-induced obesity [49]. Samuel et al. found that the G protein-coupled receptor GPR41, which is activated by short-chain fatty acids (SCFAs), can modulate the effects of gut microbiota on host adiposity [50].

The causal relationship between gut microbiota and obesity was further studied using microbiota transfer. Bäckhed et al. were the first to demonstrate that transferring normal cecal microbiota from conventional rodents resulted in increased body fat deposition and insulin resistance despite reduced food intake [48]. Turnbaugh et al., in line with previous indications, suggest that the microbiome from an obese model helps to store a greater amount of energy from the diet and that this trait is transferable through feces—microbiota transplantation [51]. Experimental studies have shown that specific commensal bacteria and their metabolites can attenuate hepatic fibrosis by restoring intestinal barrier integrity, modulating inflammation, and inhibiting hepatic stellate cell activation along the gut–liver axis [52]. A recent study transplanted human gut microbiota into rodent models, where specific germ-free rodent models (pretreated with antibiotics) were colonized with microbiota from patients with non-alcoholic steatohepatitis and healthy individuals. Following a high-fat, high-fructose diet, the rodents gained weight and exhibited more adipose tissue, hepatic steatosis, increased total cholesterol, and insulin resistance [53].

Gut microbiota transplantation into germ-free mice has also been used to assess the causal role of microbiota composition in susceptibility to nonalcoholic steatohepatitis. Le Roy et al. demonstrated that intestinal microbiota composition determines the development of NAFLD in the C57BL/6 mouse strain. By transplanting gut microbiota from mice with or without NAFLD into germ-free mice, they showed that the tendency to develop features of NAFLD, including hyperglycemia and steatosis, is transmissible via the microbiota. Furthermore, they found that the gut microbiota influences hepatic lipid metabolism independently of obesity [54]. Henao-Mejia et al. studied inflammasome-deficient mouse models to investigate the possible role of the microbiome in NAFLD [42]. They reported that the gut exhibits changes in the microbiota due to the deficiency of NLRP6 and NLRP3 inflammasomes, a deficiency that was associated with worsening hepatic steatosis and increased expression of TNF-α.

This evidence provides insight and a deeper understanding of the role of the gut microbiome in the development and progression of MASLD, as well as the mechanisms involved. These results also raise the question of the extent to which the gut microbiota plays a role in the pathogenesis of MASLD in humans and which bacteria are involved.

There are a growing number of studies revealing the association of gut microbiota dysbiosis with intestinal disorders (irritable bowel syndrome, inflammatory bowel disease, etc.) and non-intestinal disorders (metabolic syndrome, cancers, brain pathologies, etc.). In several human and animal studies, dysbiosis has been associated with both the presence and severity of MASLD [55]. Spencer et al. revealed that varying levels of Erysipelotrichia and Gammaproteobacteria were correlated with liver fat accumulation. They demonstrated that higher baseline levels of Erysipelotrichia were associated with a higher risk of developing non-alcoholic steatohepatitis, whereas higher baseline levels of Gammaproteobacteria correlated with a lower risk of developing steatosis [56]. In a study comparing the gut microbiome of patients with NAFLD and healthy controls, Gram-negative bacteria were found to be more abundant in patients with NAFLD, with up to 20% more Bacteroidetes and 24% less Firmicutes compared to healthy non-obese adults [57]. Increases in Gram-negative bacteria have also been associated with NAFLD in children. Mikhail et al. identified higher levels of epsilon-proteobacteria and gamma-proteobacteria in children with NAFLD compared to normal-weight children and healthy obese children. Moreover, children with NAFLD showed higher levels of *Prevotella* [58].

Recently, the composition of the gut microbiota was characterized by whole-genome sequencing of stool samples to differentiate between cases of mild/moderate steatosis and fibrosis [59]. Firmicutes and Proteobacteria were observed at different concentrations in these groups. Firmicutes were more commonly found in mild to moderate steatosis, whereas Proteobacteria were associated with fibrosis. Finally, these authors established a random classification model based on microbiome analysis, which possessed a strong diagnostic accuracy of advanced fibrosis [59]. Thus, human studies reveal measurable differences in gut microbiota between healthy individuals and patients with MASLD, with specific compositional changes linked to disease progression and metabolic dysfunction [60].

### 3.4. Gut Microbiota-Derived Metabolites in the Pathogenesis of MASLD and MASH

MASLD and its progressive form, MASH, are increasingly recognized as systemic metabolic disorders in which the gut microbiota and its metabolites play a central pathogenic role. Alterations in microbial composition and function (dysbiosis) influence hepatic lipid metabolism, immune responses, and intestinal barrier integrity. Among the most studied microbiota-derived metabolites involved in MASLD are SCFAs, choline-derived metabolites such as trimethylamine (TMA) and trimethylamine-N-oxide (TMAO), endogenous ethanol, bile acids, and amino acid-derived metabolites, especially those originating from tryptophan metabolism.

Short-chain fatty acids, mainly acetate, propionate, and butyrate, are produced through bacterial fermentation of dietary fibers in the colon. Under physiological conditions, SCFAs maintain intestinal barrier integrity, regulate immune responses, and influence energy homeostasis, lipid metabolism, and gluconeogenesis. Experimental and clinical studies have shown that alterations in SCFA production are associated with metabolic disorders, including MASLD. For example, reduced butyrate-producing bacteria and lower fecal butyrate levels have been observed in patients with metabolic syndrome and fatty liver disease, correlating with increased intestinal permeability and systemic inflammation [60,61,62]. Mechanistically, SCFAs interact with G-protein coupled receptors such as FFAR2 (GPR43) and FFAR3 (GPR41) to influence insulin sensitivity, lipid metabolism, and energy balance. For example, butyrate has been shown to ameliorate hepatic steatosis and improve lipid metabolism through activation of the GPR41/43-CaMKII/HDAC1-CREB signaling pathway in rodent models of diet-induced MASLD, implicating both receptor-mediated effects and epigenetic regulation of metabolic genes [61,63]. In experimental high-fat diet models, reduced SCFA production is associated with worsened steatosis, increased endotoxemia, and hepatic inflammation [62].

Choline metabolism represents another important microbiota-dependent pathway involved in MASLD pathogenesis. Dietary choline and carnitine are converted by intestinal bacteria into TMA, which is subsequently oxidized in the liver by flavin-containing monooxygenase 3 (FMO3) into TMAO. Elevated circulating TMAO levels have been associated with insulin resistance, atherosclerosis, and more severe liver steatosis in human studies. Moreover, choline-deficient diets are well-established experimental models for inducing hepatic steatosis, and dysbiosis can enhance microbial choline utilization, further reducing host choline availability and promoting liver fat accumulation [62,64].

Another proposed mechanism involves endogenous ethanol production by certain gut bacteria. Increased intestinal ethanol production has been described in patients with MASLD, particularly those with small intestinal bacterial overgrowth or dysbiosis. Ethanol and its metabolite acetaldehyde can disrupt tight junctions, increase intestinal permeability, and induce oxidative stress and inflammatory signaling in hepatocytes [62,65]. Although this mechanism is not considered the primary driver of disease, it may act as an additional factor contributing to hepatic injury within the multiple-hit model of MASLD.

Amino acid metabolism by gut microbes, especially tryptophan, is another important contributor to MASLD pathogenesis. Tryptophan is metabolized into several indole derivatives, including indole, indole-3-propionic acid, indole-3-acetic acid, and indole-3-carboxaldehyde, which can activate host receptors such as the aryl hydrocarbon receptor (AHR). Through AHR signaling, these metabolites regulate immune responses, epithelial barrier integrity, oxidative stress, and lipid metabolism [66,67]. Reduced levels of beneficial tryptophan-derived metabolites have been associated with metabolic disorders and hepatic steatosis, while supplementation with certain indoles in experimental models attenuates hepatic inflammation and lipogenesis [66,68]. In addition, disturbances in other amino acids, including branched-chain amino acids and glutamine, may contribute to insulin resistance, oxidative stress, and hepatic lipid accumulation. Recent Mendelian randomization analyses have identified metabolites such as cysteine-glutathione disulfide and 3-indoleglyoxylic acid as potential mediators of the effects of gut microbes on MASLD risk [69].

In addition to tryptophan-derived metabolites, ammonia has emerged as another microbiota-derived amino acid metabolite with potential relevance to metabolic liver disease. Produced through bacterial urease activity and amino acid deamination, excessive intestinal ammonia has been shown to promote oxidative stress, mitochondrial dysfunction, hepatic stellate cell activation, and fibrosis, suggesting that hyperammonemia may actively contribute to MASLD progression rather than simply represent a consequence of advanced liver dysfunction [70,71,72]. Other gut microbial amino acid metabolites, including p-cresol, indican (the precursor of indoxyl sulfate), and quinolinic acid, have also been described in the literature; however, their specific contribution to MASLD remains insufficiently characterized and requires further investigation.

Bile acids (BAs) are synthesized in the liver, secreted into the intestine, where they are modified by gut microbes via deconjugation, 7α-dehydroxylation, oxidation/epimerization, etc., to form secondary bile acids. The composition of the BA pool (primary vs. secondary, conjugated vs. unconjugated, hydrophobic vs. hydrophilic) is crucial because BAs act as signaling molecules via receptors such as farnesoid X receptor (FXR) and the G protein-coupled receptor TGR5 [62,73]. In MASH/MASLD, dysbiosis results in reduced conversion of primary to secondary BAs, changes in conjugation ratios and accumulation of certain deleterious BA species [62,73]. Under-activation of FXR/TGR5 signaling leads to decreased regulation of lipogenesis, reduced fatty acid oxidation, impaired glucose homeostasis, and increased inflammation. For example, reduced FXR activity tends to increase CYP7A1 expression (rate-limiting in BA synthesis), increasing potentially toxic BA burden and lipid accumulation [62]. In addition, bile acids also affect immune regulation: in NASH, there is activation of Kupffer cells, macrophages, and T cell subsets (e.g., Th17), which may be influenced by BA pool composition. Hydrophobic BAs tend to be more damaging and proinflammatory [62,73]. Therapeutically, modification of BA signaling (via FXR agonists or TGR5 ligands), or modifying gut microbiota to shift BA pools are promising. For example, in a mouse model using FGF21 (fibroblast growth factor 21) treatment, improvement in NAFLD/NASH was partially mediated by restoration of BA metabolism via microbiota changes [74].

Overall, these findings highlight the complex interplay between microbial metabolites and host metabolic pathways. Rather than acting through a single mechanism, microbiota-derived metabolites influence multiple processes simultaneously, including intestinal permeability, immune activation, oxidative stress, and hepatic lipid metabolism. This integrated perspective supports the concept of MASLD as a disease driven by multiple parallel hits, with the gut microbiota and its metabolic products representing central therapeutic targets.

## 4. Variations in the Specific Bacteria Associated with MASH

### 4.1. Phylum-Level Changes

In the human intestine, there are two dominant phyla of bacteria, Bacteroidetes and Firmicutes, which represent 90% of the intestinal microbiota, followed to a lesser extent by Actinobacteria, Proteobacteria, Fusobacteria, and Verrucomicrobia [75]. Studies in animal models have shown that high-fat diets can induce gut dysbiosis characterized by an increased abundance of Firmicutes and Proteobacteria compared with control diets [76]. Another study comparing the gut microbiome of normal-weight subjects and patients with NAFLD reported an increase in Bacteroidetes and a decrease in Firmicutes, with enrichment in Gram-negative bacteria [77]. Differences in microbiota composition between humans and animals could be due to differences in the type of fat consumed and in fat absorption mechanisms. In another human study, the abundance of Firmicutes and Bacteroidetes was similar between obese subjects and patients with NAFLD, with higher levels of Proteobacteria observed in patients with NAFLD [65]. Furthermore, these bacteria are implicated in the integrity of the intestinal barrier, which can initiate mucosal inflammation and ultimately lead to additional hepatotoxic events.

In addition, Lentisphaerae, a phylum with low representation in the gut microbiota, is observed to be decreased in patients with steatohepatitis compared with healthy patients [78]. Finally, Fusobacteria (a phylum with several bacterial pathogens, along with Proteobacteria) was increased by approximately 2.76% in patients with NAFLD, also causing increased levels of intestinal toxins caused by bacteria [79].

### 4.2. Family and Gender Variations

In a study of rodents fed a high-fat diet, an increase in the genera *Barnesiella* and *Roseburia* and a decrease in the genus *Allobaculum* was observed [54]. A clinical study compared the gut microbiome of 30 patients with NAFLD to 30 healthy subjects and found higher levels of Lactobacillaceae and Lachnospiraceae, but lower levels of Ruminococcaceae in patients with NAFLD [79].

At the genus level, *Lactobacillus*, *Dorea*, *Robinsoniella*, and *Roseburia* were over-expressed, whereas *Oscillibacter* was under-represented in the same patients with NAFLD [79]. These results were partially supported by a prospective cross-sectional study including 39 patients with biopsy-proven NAFLD, noting that the family Lactobacillaceae and the genus *Lactobacillus* were increased in patients compared to healthy controls, while levels of the genera *Coprococcus* and *Ruminococcus* decreased [80]. However, in obese children with NAFLD, an increase in the family Prevotellaceae was observed due to higher levels of the genus *Prevotella* [58]. A study revealed that the Enterobacteriaceae family and the *Escherichia* genus were more abundant in the microbiota of patients with NAFLD than in obese subjects [65].

Diet is a key factor influencing the level of *Bacteroides* and *Prevotella* in the gut: a Western diet is favorable for *Bacteroides,* while diets based on vegetables, fruits, and high-fiber foods are favorable for *Prevotella* [77]. Moreover, in advanced stages of fibrosis, the genus *Ruminococcus* increased significantly. Within this genus, there are alcohol-producing species that could lead to additional deleterious effects on intestinal permeability and liver inflammation [77].

### 4.3. Variations in Specific Bacteria Associated with Nonalcoholic Steatohepatitis

#### 4.3.1. *Faecalibacterium prausnitzii*

*Faecalibacterium prausnitzii* is a butyrate-producing bacterium [81]. It fulfills multiple roles, among which are anti-inflammatory effects and improvement of intestinal barrier function [82]. This bacterium represents >5% of the total intestinal microbiota in healthy subjects [81]. In humans, *F. prausnitzii* reduction was observed to be associated with >5% liver fat content and increased adipose tissue inflammation, which may contribute to worsening steatohepatitis [83]. These findings were supported by studies in rodents fed a high-fat diet, noting that animals treated with *F. prausnitzii* as a probiotic showed lower liver lipid content and lower plasma levels of liver transaminases, suggesting a healthier liver compared to untreated controls [84].

#### 4.3.2. *Bilophila wadsworthia*

*B. wadsworthia* is a Gram-negative proteobacterium associated with a high-fat diet [85]. It metabolizes sulfated compounds and produces hydrogen sulfide that promotes direct inflammation and damages the intestinal barrier [85] and, consequently, an increased abundance of *B. wadsworthia* is associated with increased intestinal inflammation. In addition, a study demonstrated that liver lipid and triglyceride content increased in rodents fed a high-fat diet and supplemented with *B. wadsworthia* compared to controls, which weakens liver function and potentiates the onset of metabolic syndrome [86]. Like other Gram-negative bacteria, *B. wadsworthia* can release lipopolysaccharides as endotoxins, stimulating a systemic inflammatory response, thereby increasing circulating levels of key cytokines such as serum amyloid A and interleukin-6 (IL-6) [85]. In addition, *B. wadsworthia* is involved in decreasing butyrate metabolism, which disrupts the integrity of tight junctions in the intestinal barrier. This allows lipopolysaccharides from the intestinal lumen to enter the portal vein, reach the liver, and act on liver macrophages, thus increasing the release of cytokines [86]. Finally, *B. wadsworthia* promotes a reduction in the production of primary bile acids, contributing to the disturbance of the microbiota [85].

#### 4.3.3. *Helicobacter pylori*

*Helicobacter pylori* is a Gram-negative bacillus that represents, in humans, a key factor in the etiology of various gastrointestinal diseases. Several meta-analyses have reported a significant association between *H. pylori* infection and the risk of non-alcoholic fatty liver disease. For example, pooled analyses indicate that infected individuals have approximately a 20–30% higher risk of NAFLD compared to uninfected subjects, with stronger associations observed in more advanced disease stages [87]. In addition, *H. pylori* infection modulates the release of several inflammatory cytokines (TNF-α and some interleukins, IL-1β, IL-6 and IL-8), which play an important role in hepatocellular carcinoma associated with NAFLD [88]. In addition, leptin release from adipose tissue is induced by *H. pylori* infection [88]. Leptin is a key adipokine that contributes through its role in the regulation of glucose, energy homeostasis and lipid metabolism. Increased leptin levels activate hepatic stearoyl CoA desaturase, thereby accelerating VLDL formation and fat deposition in the liver [88]. Finally, *H. pylori* infection has the greatest impact on the homeostasis of the upper digestive tract, affecting the gut–liver axis. This bacterium can increase intestinal mucosal permeability and cause dysbiosis, thus stimulating the passage of bacterial endotoxins to the liver via the portal circulation [88]. These endotoxins trigger the release of pro-inflammatory cytokines such as TNF-α and IL-8 through TLRs, leading to the migration of neutrophils and monocytes and increasing lipid accumulation in the liver [88].

#### 4.3.4. *Klebsiella pneumoniae*

In a state of homeostasis, the microbiota constantly produces ethyl alcohol in the gut, which is normally metabolized in the liver by alcohol dehydrogenase (ADH) and other liver enzymes. When the gut microbiota is enriched in alcohol-producing bacteria, alcohol production is increased, exceeding the liver’s detoxification capacity. This produces a constant supply of reactive oxygen species (ROS) in the liver, which induces liver inflammation and often results in steatohepatitis. More bacterial species with higher alcohol-producing capacity were shown in patients with NAFLD than in control patients [86]. Under both aerobic and anaerobic conditions, *K. pneumoniae* induced higher blood alcohol concentrations in patients with steatohepatitis than in controls, due to its greater alcohol-producing capacity [89].

Furthermore, after the initiation of a reduction in the abundance of *K. pneumoniae*, a dual effect was observed: a loss of body weight and a decrease in the endogenous production of alcohol by the fecal flora, suggesting an association between the presence of *K. pneumoniae* and the progression of steatohepatitis [89]. In rodents, transplantation of *K. pneumoniae* is sufficient to induce steatohepatitis, increasing serum triglyceride and transaminase concentrations. In rodents with *K. pneumoniae*-induced steatohepatitis, higher alcohol concentrations are reported in the portal vein than in the peripheral veins, demonstrating alcohol production by the microbiome [89].

#### 4.3.5. *Akkermansia muciniphila*

*Akkermansia muciniphila*, a Gram-negative bacterium from the phylum *Verrucomicrobia*, is one of the most abundant microorganisms in the human intestinal microbiota, representing between 3–5% of the entire bacterial community [90]. It is considered a bacterium with beneficial effects, which could serve as a potential probiotic treatment [43]. This bacterium is found in the mucous layer of the intestine, with mucin-degrading activity [88], and colonizes the intestine during the first month of life. In a rodent study, *A. muciniphila* is less abundant in obese animals with non-alcoholic steatohepatitis than in their counterparts [43]. This decrease is also inversely correlated with increased body weight, inflammation, insulin and glucose resistance [43]. Additionally, a thinner intestinal mucosal layer was observed in obese animals, causing increased intestinal permeability and allowing bacterial compounds to enter the circulatory system [91]. Consequently, a higher presence of *A. muciniphila* leads to improvements in metabolic parameters, lowering cholesterol levels and hepatic steatosis [43]. Metformin, widely used as a first-line antidiabetic treatment, has been shown to improve glucose levels in patients with an increased population of *A. muciniphila* [43]. Strengthening the intestinal barrier has been linked to increased circulating levels of endocannabinoids and intestinal peptides, due to the presence of *A. muciniphila* activity, which modulates the thickness of the mucus layer and promotes antimicrobial peptides and immunity [43].

*A. muciniphila* is able to obtain carbon, energy and nitrogen sources from mucin and then releases free sulfate from mucin fermentation [91]. A study by S. Zhao et al. including pathogen-free rodent models, described improvement in metabolic profiles due to daily gavage supplementation of *A. muciniphila*. This finding leads to an improvement in glucose tolerance and insulin sensitivity in the liver, through a significant reduction in the expression levels of genes involved in glucose metabolism (phosphoenolpyruvate carboxykinase and glucose-6-phosphatase) [92]. In addition, a significant reduction in the expression levels of hepatic genes involved in fatty acid synthesis (SREBP1c) and transport (translocase acid (CD36)) was observed due to *A. muciniphila* supplementation, leading to less fat deposition. In addition, plasma lipopolysaccharide-binding protein binds lipopolysaccharides, facilitating their recognition by the TLR4 receptor, leading to downstream signaling that results in inflammation [93]. Levels of lipopolysaccharide-binding proteins were reduced in the systemic circulation by the increased presence of *A. muciniphila*, reducing metabolic endotoxemia and downstream signaling. These metabolic benefits induced by *A. muciniphila* could offer possibilities to prevent or ameliorate different disorders.

## 5. Gut Microbiota Signatures Associated with MASLD

Recent research has increasingly highlighted the role of the gut microbiota in the pathogenesis and progression of pediatric MASLD (Table 1). Several studies have demonstrated that children with MASLD exhibit distinct alterations in gut microbiota composition and function compared to healthy peers or obese children without liver disease. For example, Yu et al. found that pediatric NAFLD is associated with reduced levels of secondary bile acids and a lower abundance of bacteria such as *Eubacterium* and members of the family Ruminococcaceae, which are involved in bile acid metabolism. This study suggested that a dysfunctional conversion of primary to secondary bile acids may contribute to disease development through altered host–microbe interactions and impaired gut–liver axis signaling [94]. Similarly, Zhao et al. reported that children with NAFLD had increased levels of Proteobacteria, particularly Gammaproteobacteria, and reduced abundance of beneficial genera like *Alistipes*. These microbial changes were accompanied by alterations in metabolic pathways, including those involved in amino acid and glycan metabolism, indicating both taxonomic and functional dysbiosis in MASLD [95]. In a study by Schwimmer et al. [96], the microbiome of children with more severe liver disease—namely steatohepatitis and moderate to severe fibrosis—contained higher levels of genes associated with lipopolysaccharide (LPS) synthesis and flagellar assembly, which are known to contribute to inflammation. These findings point to a potential microbial contribution to disease severity through pro-inflammatory microbial products [96]. Another pediatric study highlighted the reduction in key beneficial bacteria, including *Akkermansia* and *Bacteroides*, in children with NAFLD. This microbial signature was accompanied by a distinct beta-diversity profile when compared to healthy controls, suggesting that even in young children, the gut microbiota may serve as a marker of liver health [97]. Further supporting the microbiome–NAFLD connection, the EPOCH study found that gut microbial diversity was inversely associated with hepatic fat accumulation in adolescents. Specific taxa such as *Bilophila* and *Paraprevotella*, combined with dietary intake and BMI, were significantly correlated with liver fat levels, highlighting how diet, obesity, and microbiota interact in early NAFLD pathogenesis [98]. A broader review by Zhu et al. [99] reported that children with NAFLD frequently exhibit reductions in SCFA-producing bacteria, increased intestinal permeability, and endotoxin translocation. These mechanisms may contribute to hepatic inflammation and steatosis, highlighting the gut–liver axis as a potential therapeutic target in pediatric MASLD [99].

## 6. Transition from NAFLD to MAFLD and MASLD: An Evolving Metabolic Paradigm

In recent years, the terminology surrounding fatty liver disease has undergone substantial revision, reflecting advances in the understanding of its pathophysiology. The traditional term NAFLD was introduced to describe hepatic steatosis not caused by significant alcohol intake. However, this definition has been increasingly criticized as exclusionary, not accounting for metabolic dysfunction as the central driver, and creating ambiguity in patients with mixed etiologies [100]. In 2020, an international expert consensus proposed the term MAFLD, shifting toward a positive diagnostic framework based on evidence of metabolic dysregulation [101]. Although introduced in 2020, the clinical and epidemiological validation of MAFLD has largely occurred in studies published after 2021 [101].

Several post-2021 studies demonstrated that the MAFLD definition better identifies patients at higher risk of advanced fibrosis and cardiovascular disease compared with NAFLD criteria [102,103]. For example, Fu et al. showed that patients fulfilling MAFLD criteria had a higher risk of all-cause and cardiovascular mortality compared to those classified under NAFLD without metabolic dysfunction [104].

In 2023, a multi-society Delphi consensus further refined the nomenclature, introducing the term metabolic dysfunction-associated steatotic liver disease (MASLD) as an umbrella definition and metabolic dysfunction-associated steatohepatitis (MASH) for the inflammatory subtype [105]. This updated terminology preserves the metabolic core introduced by MAFLD while harmonizing definitions globally and addressing concerns raised during the MAFLD debate. Collectively, the transition from NAFLD to MAFLD—and subsequently to MASLD—represents a paradigm shift:

- from an exclusion-based diagnosis to a metabolic dysfunction-driven definition
- from a liver-centric concept to a systemic metabolic disease model
- from descriptive terminology to one aligned with mechanistic understanding and risk prediction

This conceptual evolution has important implications for microbiota research, as metabolic dysfunction, insulin resistance, bile acid signaling, and inflammatory pathways—central to MAFLD/MASLD—are also key axes in gut–liver interactions.

## 7. Transition from Classical Microbiota Studies to Metagenomic Approaches in MASLD

Early investigations into the gut microbiota in MASLD were primarily based on 16S rRNA sequencing techniques and focused on broad taxonomic shifts, such as changes in the Firmicutes/Bacteroidetes ratio or variations in a limited number of dominant genera. Although these studies provided important initial insights, their findings were frequently inconsistent. These discrepancies likely reflect differences in study populations (children versus adults), disease stage, obesity status, dietary habits, ethnicity, geographical background, and methodological factors, including DNA extraction protocols, sequencing platforms, bioinformatic pipelines, and the use of 16S rRNA sequencing rather than shotgun metagenomics. Collectively, these limitations have highlighted the restricted functional resolution of taxonomic profiling alone and have driven the transition toward metagenomic and multi-omics approaches capable of characterizing microbial genes, metabolic pathways, and host–microbiota interactions.

Recent research has increasingly shifted toward metagenomic and multi-omics approaches, allowing a more comprehensive characterization of microbial genes, metabolic pathways, and host–microbiota interactions. Beyond describing taxonomic alterations, the metagenomic studies have increasingly focused on the functional capacity of the gut microbiome, providing insights into the microbial genes and metabolic pathways that may contribute to MASLD pathogenesis. In contrast to 16S rRNA sequencing, shotgun metagenomics enables simultaneous characterization of microbial taxonomic composition and functional gene content, allowing reconstruction of metabolic pathways involved in host–microbiota interactions. This functional approach has demonstrated that disease-associated microbial signatures extend beyond changes in bacterial abundance and include alterations in pathways related to microbial metabolism that correlate with liver injury and fibrosis.

A contemporary review by Tilg et al. [106] emphasized that NAFLD pathogenesis is driven not only by compositional dysbiosis but also by functional alterations, including enhanced microbial ethanol production, endotoxin-mediated inflammation, and disrupted bile acid signaling. These functional disturbances are now considered central components of the gut–liver axis [106].

At a mechanistic level, Quesada-Vázquez et al. [93] highlighted how diet-induced dysbiosis influences hepatic lipid metabolism through several interconnected pathways, including increased energy harvest from the diet, disturbances in choline metabolism leading to trimethylamine production, and modulation of bile acid–FXR signaling. These mechanisms provide a functional explanation for the association between Western dietary patterns, gut dysbiosis, and NAFLD progression [93].

A landmark study by Loomba et al. demonstrated that a gut microbiome–derived metagenomic signature could non-invasively identify advanced fibrosis in patients with biopsy-proven NAFLD. This study identified enrichment of Proteobacteria, including *Escherichia coli*, and depletion of beneficial taxa in patients with advanced fibrosis, establishing proof-of-concept for microbiome-based diagnostic models [59].

Subsequently, Caussy et al. integrated untargeted serum metabolomics with shotgun metagenomic sequencing in a validation subgroup of patients with biopsy-proven NAFLD to investigate host–microbiota interactions associated with hepatic steatosis and fibrosis. Using a twin-based genetic model, the authors identified several circulating metabolites sharing genetic effects with both steatosis and fibrosis, among which the gut microbiota-derived metabolite 3-(4-hydroxyphenyl) lactate emerged as the most consistent finding across the discovery and validation cohorts. Pathway reconstruction further linked this metabolite to specific gut microbial species, providing proof-of-concept that integrating metagenomic and metabolomic data can uncover functional host–microbiota relationships associated with MASLD progression [107].

Recent pediatric multi-omics studies have begun to complement taxonomic analyses with metabolomic profiling. For example, Du et al. demonstrated that children with MASLD exhibit reduced gut microbial diversity and that enrichment of Ruminococcus torques is associated with increased liver stiffness and elevated circulating deoxycholic acid (DCA), highlighting the potential of integrating microbial and metabolic signatures to better characterize disease-associated host–microbiota interactions. These findings illustrate how integrating microbiome sequencing with metabolomic profiling may provide functional biomarkers beyond microbial taxonomy alone [108].

Furthermore, the first meta-analysis of shotgun metagenomic sequencing in pediatric MASLD and MASH expanded the characterization of the gut microbiome beyond taxonomic composition by integrating species abundance with microbial gene and metabolic pathway analyses. The authors demonstrated that both gene- and pathway-abundance profiles accurately discriminated pediatric MASLD and MASH from obesity using machine-learning models. Functional analyses further revealed reduced abundance of pathways involved in butyrate and propanoate synthesis, together with alterations in bacterial bile acid synthesis, providing mechanistic insight into microbial metabolic dysfunction associated with pediatric MASLD. These findings support the growing role of functional metagenomics in identifying biologically relevant pathways that may complement conventional taxonomic profiling and facilitate future biomarker discovery [109].

More recently, Lin et al. integrated shotgun metagenomics with transcriptomic and metabolomic analyses to characterize functional host–microbiota interactions in NAFLD. Their multi-omics approach identified coordinated alterations in microbial metabolic pathways—including tryptophan, branched-chain amino acid, glycogen degradation, and bile acid metabolism—and linked these changes to differential host gene expression. By reconstructing gene–microbiota–metabolite interaction networks, the study demonstrated how integrative multi-omics analyses can provide mechanistic insight into the metabolic processes underlying NAFLD beyond conventional taxonomic profiling [110].

Overall, these modern metagenomic and multi-omics studies indicate that MASLD is characterized less by a single disease-specific microbial composition and more by reproducible functional alterations, including reduced short-chain fatty acid production, enrichment of endotoxin-producing bacteria, disturbances in bile acid metabolism, and shifts in microbiota-derived metabolites. This transition from purely taxonomic analyses to functional and integrative approaches represents a major advance in understanding the gut–liver axis and may facilitate the development of microbiota-based diagnostics and targeted therapies in MASLD.

## 8. Microbiota-Targeted Therapies in MASLD and Emerging Pharmacological Approaches Interacting with the Gut–Liver Axis

### 8.1. Probiotics, Prebiotics and Synbiotics

Probiotics are live microorganisms that, when administered in adequate amounts, have been shown to confer health benefits by restoring microbial balance, enhancing intestinal barrier integrity, and reducing systemic inflammation. Several randomized controlled trials and meta-analyses have suggested that probiotic supplementation in MASLD patients can reduce liver enzymes, improve markers of inflammation, and reduce hepatic steatosis and liver stiffness [111,112]. Mechanistically, probiotics may strengthen the gut barrier, decrease endotoxin translocation, modulate immune responses, and increase beneficial metabolites such as short-chain fatty acids, which can exert anti-inflammatory and metabolic effects [113]. However, pediatric evidence remains limited, with relatively few randomized studies, small sample sizes, heterogeneous probiotic formulations, and inconsistent clinical outcomes. Consequently, current pediatric guidelines do not recommend probiotics as standard therapy for MASLD, although they remain a promising area for future investigation.

Prebiotics are non-digestible food components (e.g., inulin, fructo-oligosaccharides) that selectively stimulate the growth or activity of beneficial gut bacteria. Prebiotic fermentation by gut microbes produces SCFAs such as propionate and butyrate, which have been shown to inhibit hepatic lipogenesis and reduce fat accumulation in the liver. Clinical and experimental studies suggest prebiotics can improve gut barrier integrity, reduce systemic endotoxemia, and thereby lessen inflammation and metabolic stress associated with MASLD [114]. Nevertheless, clinical evidence in children remains insufficient to support routine use, and larger well-designed pediatric trials are required before firm recommendations can be made.

Synbiotics combine probiotics and prebiotics in a single intervention, aiming to both introduce beneficial microbes and provide substrates that support their growth. Clinical evidence suggests that synbiotic treatment can lead to significant reductions in liver enzymes, hepatic steatosis, and measures of fibrosis, sometimes with superior effects compared to probiotics alone. The combined approach may enhance colonization and metabolic activity of beneficial bacteria, further improving gut–liver axis signaling [112]. However, although preliminary studies have reported encouraging results, the available pediatric evidence is limited and does not yet support routine clinical use outside research settings.

Overall, microbiota-targeted therapies represent an attractive therapeutic strategy because they address one of the key mechanisms implicated in MASLD pathogenesis. Nevertheless, current evidence in children remains limited, and most available data originate from small randomized trials or extrapolation from adult studies. Therefore, probiotics, prebiotics, synbiotics, and other microbiota-directed interventions should currently be regarded as promising investigational approaches rather than established standard therapies.

### 8.2. Resmetirom: An Approved Pharmacological Therapy for Adult MASH with Potential Indirect Interactions with the Gut–Liver Axis

Resmetirom is a liver-directed, selective thyroid hormone receptor-β (THR-β) agonist recently approved for the treatment of non-cirrhotic MASH with fibrosis in adults. It is designed to reproduce the beneficial metabolic actions of endogenous thyroid hormone specifically within hepatocytes, where THR-β is the predominant receptor isoform, thereby minimizing systemic thyrotoxic effects. Activation of hepatic THR-β increases mitochondrial β-oxidation, enhances lipid mobilization, and reduces de novo lipogenesis, leading to decreased intrahepatic triglyceride accumulation, improved lipid profiles, and reduced lipotoxicity [115,116,117,118,119].

**Figure 1:**
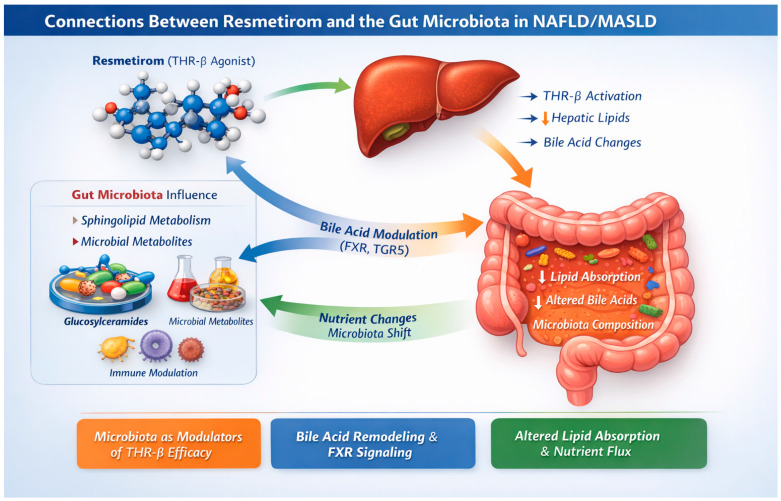
Hypothetical indirect interactions between resmetirom therapy and the gut–liver axis based predominantly on experimental and preclinical evidence—As a selective hepatic THR-β agonist, resmetirom enhances lipid metabolism and reduces hepatic fat while altering bile acid synthesis and composition. These changes influence intestinal lipid absorption and microbial ecology. In turn, the gut microbiota modulates drug efficacy through microbial metabolites, sphingolipid pathways, immune signaling, and bile acid-dependent FXR/TGR5 pathways, forming a feedback loop within the gut–liver axis that may affect therapeutic response.

While resmetirom does not directly target the gut microbiota, several biologically plausible mechanisms suggest that THR-β activation may indirectly influence gut–liver axis signaling through changes in bile acid metabolism and host–microbiota interactions (Figure 1).

#### 8.2.1. Modification of Bile Acid Profiles

Bile acids are primary signaling molecules between the liver and gut microbiota: they regulate FXR and TGR5 signaling pathways that influence both host metabolism and microbial ecology [120]. Although resmetirom does not have a direct anti-microbiota action, it modulates bile acid profiles by increasing bile acid synthesis and altering lipid absorption patterns. Changes in bile acid pools induced by resmetirom can shift the balance between primary and secondary bile acids—metabolites largely produced by gut bacteria—which can influence microbial community structure. This bile acid modulation could indirectly select for or against certain microbial populations (e.g., those specialized in bile acid transformation), potentially influencing gut barrier function, inflammation, and metabolic signaling [121]. While resmetirom’s effects on intestinal bile acids have been shown mainly in rodent models, this opens a plausible link between THR-β agonism, bile acid signaling, and the microbiome.

#### 8.2.2. Potential Effects on Gut Lipid Absorption

One preclinical study showed that THR-β agonism reduced intestinal lipid absorption by altering lipid and bile acid compositions. Altered lipid absorption can change the nutrient availability in the intestine, shaping microbial ecology by influencing which substrates reach the colon. Microbial communities are highly sensitive to the types of fats and bile acids that reach them; changes in these can shift community composition, metabolism, and metabolite production—which, in turn, affect liver inflammation and metabolic phenotypes [121].

#### 8.2.3. Metabolic Intermediates and Host–Microbe Crosstalk

Emerging reviews on MASLD therapies emphasize that drugs like resmetirom may interact with host bile acid–FXR–FGF19 signaling and systemic metabolism in ways that depend on gut microbial metabolites. The altered FXR signaling can modify FGF19 release, which is a hormone that influences both hepatic lipid metabolism and intestinal function—and whose regulation is intertwined with microbiota-derived bile acid metabolites [122]. While this is still hypothetical, it suggests a network of interdependent signals between resmetirom action, bile acid signaling pathways, and microbial metabolite production.

#### 8.2.4. Microbiota as a Modulator of THR-β Agonist Efficacy

A recent study investigated a THR-β agonist closely related to resmetirom and found that gut microbiota composition significantly influenced drug efficacy in MASH models. In mice, the THR-β agonist HSK31679 (structurally similar to resmetirom) was more effective in ameliorating steatohepatitis in the presence of an intact microbiota than in germ-free mice, suggesting that bacterial metabolism contributes to the therapeutic effect. This effect was linked to changes in microbial sphingolipid metabolism—specifically, modifications of microbial sphingolipids by bacterial glucosylceramide synthase. These microbial lipids were connected to immune regulation and inflammatory signaling relevant to liver disease [123]. This represents a clinically relevant knowledge gap that may partly explain interindividual variability in therapeutic response.

Although these observations suggest a potential interaction between resmetirom and the gut microbiota, current evidence remains predominantly preclinical, and no studies have evaluated these mechanisms in pediatric MASLD. Therefore, any microbiota-mediated effects of resmetirom should currently be regarded as hypothetical. More broadly, the clinical translation of microbiota-targeted therapies in pediatric MASLD remains challenging, as evidence regarding probiotics, prebiotics, synbiotics, and microbiome-related effects of pharmacological therapies is derived mainly from small clinical studies, heterogeneous patient populations, or experimental models. Large, well-designed randomized controlled trials in pediatric populations are required before these approaches can be routinely implemented in clinical practice.

## 9. The Bidirectional Relationship Between Gut Dysbiosis and MASLD

Despite the growing body of evidence linking gut microbiota alterations to MASLD, an important unresolved question is whether intestinal dysbiosis represents a primary driver of disease or develops as a consequence of hepatic metabolic dysfunction. Experimental studies support a causal contribution of the gut microbiota, as fecal microbiota transplantation from patients with steatohepatitis or metabolic syndrome into germ-free mice induces hepatic steatosis, insulin resistance, and inflammatory changes, suggesting that specific microbial communities can directly promote liver injury. Conversely, accumulating clinical and mechanistic evidence suggests that hepatic steatosis and the metabolic disturbances accompanying MASLD—including altered bile acid metabolism, chronic inflammation, and changes in nutrient availability—may themselves contribute to remodeling of the intestinal microbiota. Current evidence therefore supports a bidirectional relationship in which dysbiosis and liver disease reinforce each other through the gut–liver axis rather than a simple unidirectional cause-and-effect mechanism. Longitudinal human studies integrating metagenomics with metabolomics and host transcriptomics will be required to establish temporal relationships and determine whether microbial alterations precede disease onset or mainly reflect disease progression [26,38,54].

## 10. Discussion

We evaluated articles from PubMed, Scopus, and Web of Science using the following search terms—gut microbiota, dysbiosis, microbiome, pediatric MAFLD, MASLD, NAFLD, NASH, MASH, metagenomics, metabolomics, gut–liver axis, intestinal permeability, bile acids, short-chain fatty acids, probiotics, prebiotics, synbiotics, postbiotics, fecal microbiota transplantation, and microbiota-targeted therapies—and identified several studies that highlighted variations in bacterial species in patients with MASLD (Table 2).

Zhu et al. demonstrated an increased Bacteroidetes/Firmicutes ratio in children with NASH and obesity compared with healthy controls [65]. Similar results were observed in other studies; however, this finding remains controversial. For example, Monga Kravetz et al. reported a lower Bacteroidetes/Firmicutes ratio in obese youth with NAFLD, highlighting the inconsistency of phylum-level alterations across pediatric studies [124]. Zhao et al. also reported a decreased Bacteroidetes/Firmicutes ratio in obese children with NASH, reflecting a relative reduction in Bacteroidetes despite no significant changes in Firmicutes. However, given the inconsistency across pediatric studies, its value as a reliable biomarker remains uncertain [95].

In studies by Zhu et al. and Michail et al., a substantial increase in *Prevotella* was observed in patients with NAFLD compared to healthy controls [58,65]. In contrast, Monga Kravetz et al. reported low *Prevotella* levels in similar patients [124].

Other studies have focused on specific microbial taxa rather than global phylum-level ratios. Zhao et al. reported an increased abundance of Proteobacteria, especially Gammaproteobacteria, in obese children with NASH. At the same time, beneficial bacteria such as *Lactobacillus* and *Faecalibacterium prausnitzii* were reduced. Functional predictions also indicated alterations in amino acid and glycan metabolism pathways, suggesting that both taxonomic and metabolic dysbiosis may contribute to disease progression [95]. Reduced abundance of *Faecalibacterium prausnitzii* has been associated with several inflammatory and metabolic disorders, including inflammatory bowel disease, irritable bowel syndrome, and type 2 diabetes [125].

In contrast to pediatric findings, adult studies have also demonstrated microbiota alterations in MASLD, although with somewhat different microbial signatures. For instance, Wong et al. reported an increased abundance of Parabacteroides in adult patients with NASH compared to healthy controls. Notably, probiotic supplementation in this cohort did not lead to significant changes in the overall microbiota composition after six months, suggesting that microbiota modulation may be complex and influenced by multiple host and environmental factors [126].

In the study by Zhao et al., *Helicobacter pylori* and Proteobacteria are accumulated differently in obese children compared to healthy children [95]. A study conducted in China [127] revealed that *Helicobacter pylori* infection is closely related to the development of NAFLD. The eradication of this infection could represent a new adjunctive therapy for MASLD. However, the scientific literature provides discordant data regarding this hypothesis, as many studies have not found a direct connection between H. pylori infection and MASLD. Similarly, in the study by Zhao et al., no significant changes were observed between patients with NAFLD and healthy children. In this study, fecal samples were taken, whereas in most of the studies listed above, *Helicobacter pylori* detection was performed via serum antibodies or the urea test [95].

Zhu et al. demonstrated an enrichment of ethanol-producing bacteria, particularly *Escherichia coli*, suggesting a potential contribution of endogenous ethanol production to NASH pathogenesis [65]. This study is consistent with that of Michail et al., who also measured ethanol in soluble fractions and reported an increase in this metabolite in obese patients with NAFLD [58]. Although in the study carried out by Del Chierico et al., no significant differences were observed in the population of *E. coli* compared to normal controls, they revealed a higher abundance of Enterobacteriaceae in patients with NAFLD [128]. In addition, Zhu et al. highlighted that several gut bacterial genera, including *Bacteroides*, *Bifidobacterium*, and *Clostridium*, have been reported to produce endogenous ethanol, potentially contributing to NASH pathogenesis [65].

There are several studies investigating the potential role of probiotic supplementation in liver function. Among the probiotic strains investigated, *Lactobacillus rhamnosus* has shown the most encouraging results, being associated with reductions in transaminase levels and possible improvements in liver fibrosis in pediatric MASLD. Nevertheless, the available evidence is based on relatively small studies and remains insufficient for routine clinical recommendation [129]. These findings suggest that probiotics have a role in improving MASLD in pediatric patients.

Overall, these studies consistently demonstrate the presence of gut dysbiosis in MASLD, although the specific microbial signatures vary across populations and study designs. Importantly, the inconsistency in findings regarding the Bacteroidetes/Firmicutes ratio indicates that phylum-level markers alone may not adequately reflect disease-specific alterations. Instead, changes in specific bacterial taxa, functional pathways, and microbial metabolites appear to be more relevant for understanding the role of the gut microbiota in MASLD pathogenesis.

Mechanistically, gut microbiota may influence MASLD progression through several pathways, including increased intestinal permeability, endotoxin translocation, altered bile acid metabolism, and production of microbial metabolites such as ethanol, short-chain fatty acids, and amino acid derivatives. These factors can promote hepatic inflammation, insulin resistance, and lipid accumulation.

Given these findings, modulation of the gut microbiota represents a promising therapeutic target. Interventions such as probiotics, prebiotics, synbiotics, and dietary modifications have shown beneficial effects in some studies, including reductions in transaminase levels, improvements in metabolic parameters, and attenuation of hepatic steatosis. However, results remain heterogeneous, and further large, well-designed clinical trials are needed to establish the efficacy of microbiota-targeted therapies in pediatric MAFLD.

**Table 2:** Gut microbiota profiles and *Bacteroidetes*/*Firmicutes* ratio in pediatric MASLD/MASH.

References	Characteristics of Participants	*Bacteroidetes*/*Firmicutes* Ratio	Results	Notes
[65]	Participants: Children: 25 with obesity, 22 with NASH, 16 HC	Findings: ↑ in NASH and obese patients	↑ *Escherichia* ↑ *Prevotella* ↑ *Peptoniphilus* ↑ *Coprococcus* ↓ *Eubacterium* ↓ *Bifidobacterium* ↓ *Oscillospira*	Conclusion: *Escherichia* was markedly increased in NASH patients compared to obese patients.
[58]	Participants: Children: 11 with obesity, 13 with NASH, 26 HC Exclusion criteria: No antibiotics for 6 months	Findings: No significant differences between NASH and obese groups compared to HC	↑ *Prevotella* in NASH compared to healthy controls; increased fecal ethanol levels in NASH	Conclusion: *Prevotella* associated with NASH rather than obesity alone.
[126]	Participants: Adults: 16 with NASH, 22 HC Exclusion criteria: No information about antibiotic/probiotic use	Not reported	↑ *Parabacteroides* ↑ *Allisonella* ↓ *Faecalibacterium* ↓ *Anaerosporobacter*	Conclusion: NASH patients were treated with probiotics/placebo, but no significant changes in gut microbiota were observed after 6 months
[128]	Participants: 61 patients with NASH, NAFL, or obesity and 54 HC	Not specifically reported	Altered Enterobacteriaceae abundance; reduced *Oscillospira* and Rickenellaceae; distinct microbial and metabolomic signatures discriminating NAFL and NASH	Integrated meta-omics identified progressive microbiome remodeling associated with pediatric disease severity.
[124]	Participants: Children (13 ± 3 years): 44 obese with NAFLD, 29 obese without NAFLD	↓ in NAFLD patients vs. group without NAFLD	↓ *Prevotella* ↓ *Oscillospira*	Demonstrates inconsistency of B/F ratio across pediatric studies

NASH—non-alcoholic steatohepatitis; HC—healthy controls; NAFL—non-alcoholic fatty liver.

Despite the growing body of evidence linking gut microbiota alterations to MASLD, several methodological limitations must be considered when interpreting these findings. Many microbiome studies include heterogeneous cohorts with differences in: age (children vs. adults), ethnicity and geography, dietary patterns, metabolic comorbidities, and medication exposure. Because the gut microbiome is highly sensitive to environmental and lifestyle factors, these variables may confound associations between microbial composition and liver disease.

Another major limitation is the variability in diagnostic definitions of fatty liver disease. Earlier studies relied on ultrasonography, liver enzyme levels and clinical criteria, while others used liver biopsy, which remains the gold standard. In addition, the transition from NAFLD to MASLD terminology has introduced new inclusion criteria based on metabolic dysfunction, making cross-study comparisons more difficult.

In addition, studies use different microbiome profiling techniques, including: 16S rRNA gene sequencing, shotgun metagenomics or targeted metabolomics. While 16S sequencing provides information at the genus level, it lacks the resolution needed to identify species-specific or functional differences. Metagenomic approaches offer higher resolution but are more expensive and computationally complex, leading to smaller sample sizes in some studies.

On the other hand, most microbiome studies in MASLD are cross-sectional. As a result, causal relationships cannot be established, and it remains unclear whether dysbiosis is a cause or consequence of liver disease. Longitudinal and interventional studies are still relatively scarce. Many metagenomic studies involve relatively small cohorts, often fewer than 100 participants. Although some studies report high diagnostic accuracy for microbiome-based models, external validation across independent populations is frequently lacking.

Taken together, these methodological limitations suggest that:

- Microbiome alterations in MASLD are consistent at the functional level.
- However, taxonomic signatures are less reproducible across studies.
- Standardized protocols and large longitudinal cohorts are needed.

Future research should focus on harmonized diagnostic criteria, multi-omics integration (metagenomics, metabolomics, transcriptomics), longitudinal and interventional study designs, and validation across diverse populations.

## 11. Conclusions

The expanding body of evidence linking gut microbiota alterations to the development and progression of MASLD underscores the complexity of the gut–liver axis and its metabolic, inflammatory, and immunological interactions. Early microbiome studies primarily focused on taxonomic shifts, such as changes in the Bacteroidetes/Firmicutes ratio; however, more recent metagenomic and metabolomic investigations highlight the importance of functional microbial pathways, including bile acid transformation, ethanol production, short-chain fatty acid metabolism, and amino acid-derived metabolites.

An important limitation of the current evidence is the relative scarcity of pediatric-specific studies. Although adult cohorts and experimental models have substantially advanced our understanding of microbiota-mediated mechanisms in MASLD, dedicated pediatric investigations remain essential to determine whether these findings can be directly translated to children. Future multicenter prospective studies integrating clinical, metagenomic, metabolomic, and host transcriptomic data are warranted to establish age-specific biomarkers and therapeutic targets.

Although specific microbial signatures have been associated with hepatic steatosis, inflammation, and fibrosis, inter-study variability remains substantial due to differences in sequencing methodologies, population characteristics, dietary patterns, and disease definitions. Consequently, no single microbial taxon or diversity index currently qualifies as a validated clinical biomarker for MASLD severity or progression.

Advances in shotgun metagenomics and integrated multi-omics approaches have shifted the paradigm from descriptive microbiota profiling toward a mechanistic understanding of microbial–host metabolic crosstalk. This transition aligns with the evolving terminology from NAFLD to MAFLD/MASLD, emphasizing metabolic dysfunction as a central driver of disease.

Future research should prioritize longitudinal cohort studies, standardized bioinformatic pipelines, and functional validation of microbial metabolites in order to clarify causality and therapeutic potential. The microbiome represents a promising but still evolving target in the prevention and treatment of fatty liver disease, and translation into clinical practice will require rigorous validation in large, well-characterized populations. Bridging mechanistic insights with therapeutic innovation represents a key priority in the evolving landscape of pediatric MASLD research.

## Figures and Tables

**Table 1:** Gut microbiota alterations associated with pediatric non-alcoholic fatty liver disease.

Population	Main Microbiota/Functional Alteration(s) Reported	Implications/Notes	References
32 children with NAFLD, 36 healthy controls (age ~8–15 years)	Reduced levels of secondary bile acids; decreased abundance of bacteria such as *Eubacterium* and Ruminococcaceae that carry bile salt hydrolase (BSH) and 7α-dehydroxylase; negative correlation between fecal lithocholic acid (LCA) and *Escherichia coli* (which is increased)	Suggests that impaired conversion of primary → secondary bile acids may be part of the gut–liver axis in pediatric NAFLD. Potential biomarkers or therapeutic target (modulating bile acid metabolism).	[94]
Obese children with NAFLD vs. obese without NAFLD vs. healthy children	↑ Proteobacteria (especially Gammaproteobacteria) in obese NAFLD; ↓ *Alistipes* (Bacteroidetes) in NAFLD; *Faecalibacterium prausnitzii* differed between obese NAFLD vs. obese non-NAFLD; functional pathways altered (amino acid metabolism, glycan metabolism, replication/repair, etc.)	Indicates that specific taxa may distinguish NAFLD beyond obesity; functional shifts may contribute (or result from) disease.	[95]
Children with NAFLD; severity stratified by histology (steatohepatitis, fibrosis)	Higher abundance of bacterial genes involved in lipopolysaccharide (LPS) synthesis and flagellar assembly in more severe disease; disrupted microbiome vs. non-NAFLD controls	Suggests microbial products that can provoke inflammation (e.g., LPS, flagellin) may drive histologic progression; possible markers of severity.	[96]
63 children with NAFLD, 63 healthy controls (age ~6.7 years average)	Differences in β-diversity; healthy children had higher abundance of *Bacteroidetes*, *Verrucomicrobia*, *Bacteroides*, *Akkermansia*, *Peptoclostridium*, whereas children with NAFLD exhibited enrichment of *Actinobacteria*, *Collinsella*, *Escherichia*-*Shigella*, *Roseburia*, and *Bifidobacterium*.	Suggests that pediatric NAFLD is associated with gut microbial dysbiosis characterized by depletion of potentially beneficial taxa (particularly *Akkermansia*) and enrichment of bacteria linked to inflammation. The study also highlighted interactions between lymphocyte counts and gut microbiota composition.	[97]
107 adolescents from the EPOCH cohort; hepatic fat fraction measured by MRI (population-based study, not only diagnosed NAFLD); correlational with diet and microbiota	Lower α-diversity associated with higher hepatic fat; two taxa (*Bilophila* and *Paraprevotella*) plus dietary monounsaturated fat intake & BMI z-score explained ~32% of the variation in hepatic fat fraction.	Demonstrates that even among adolescents (not necessarily all with clinical NAFLD), gut microbiota + diet + obesity metrics are predictive; suggests early detection possible via microbiota signatures.	[98]

## Data Availability

No new data were created or analyzed in this study. Data sharing is not applicable to this article.

## Abbreviations

The following abbreviations are used in this manuscript:

B/F ratio	Bacteroidetes/Firmicutes ratio
BMI	Body mass index
FXR	Farnesoid X receptor
HC	Healthy controls
LPS	Lipopolysaccharide
MAFLD	Metabolic dysfunction-associated fatty liver disease
MASLD	Metabolic dysfunction-associated steatotic liver disease
MASH	Metabolic dysfunction-associated steatohepatitis
NAFL	Non-alcoholic fatty liver
NAFLD	Non-alcoholic fatty liver disease
NASH	Non-alcoholic steatohepatitis
SCFAs	Short-chain fatty acids
THR-β	Thyroid hormone receptor β
TGR5	Takeda G-protein-coupled receptor 5
